# Analysis of 3D Prints by X-ray Computed Microtomography and Terahertz Pulsed Imaging 

**DOI:** 10.1007/s11095-016-2083-1

**Published:** 2016-12-21

**Authors:** Daniel Markl, J. Axel Zeitler, Cecilie Rasch, Maria Høtoft Michaelsen, Anette Müllertz, Jukka Rantanen, Thomas Rades, Johan Bøtker

**Affiliations:** 1 Department of Chemical Engineering and Biotechnology, University of Cambridge, Philippa Fawcett Drive, Cambridge, CB3 0AS UK; 2 Department of Pharmacy, University of Copenhagen, Universitetsparken 2, 2100 Copenhagen, Denmark

**Keywords:** 3D printing, microstructure, polyvinyl alcohol (PVA), terahertz pulsed imaging (TPI), X-ray computed microtomography (XμCT)

## Abstract

**Purpose:**

A 3D printer was used to realise compartmental dosage forms containing multiple active pharmaceutical ingredient (API) formulations. This work demonstrates the microstructural characterisation of 3D printed solid dosage forms using X-ray computed microtomography (XμCT) and terahertz pulsed imaging (TPI).

**Methods:**

Printing was performed with either polyvinyl alcohol (PVA) or polylactic acid (PLA). The structures were examined by XμCT and TPI. Liquid self-nanoemulsifying drug delivery system (SNEDDS) formulations containing saquinavir and halofantrine were incorporated into the 3D printed compartmentalised structures and *in vitro* drug release determined.

**Results:**

A clear difference in terms of pore structure between PVA and PLA prints was observed by extracting the porosity (5.5% for PVA and 0.2% for PLA prints), pore length and pore volume from the XμCT data. The print resolution and accuracy was characterised by XμCT and TPI on the basis of the computer-aided design (CAD) models of the dosage form (compartmentalised PVA structures were 7.5 ± 0.75% larger than designed; *n* = 3).

**Conclusions:**

The 3D printer can reproduce specific structures very accurately, whereas the 3D prints can deviate from the designed model. The microstructural information extracted by XμCT and TPI will assist to gain a better understanding about the performance of 3D printed dosage forms.

**Electronic supplementary material:**

The online version of this article (doi:10.1007/s11095-016-2083-1) contains supplementary material, which is available to authorized users.

## Introduction

Over the last decade 3D printing of pharmaceuticals has generated growing interest in the academic community as well as in the industry given the potential of the technology as a processing platform for patient-centred dosage forms. In contrast to traditional powder compaction, 3D printing enables implementation of totally new product design principles and changes to dose and dosage form geometry can be achieved easily. The recent product launch of SPRITAM, a 3D printed orally disintegrating dosage form indicates that innovative manufacturing principles, such as 3D printing are rapidly maturing into a commercially feasible platform for drug production (1,2). In addition to the new opportunities in the field of patient-centred medicine it was demonstrated that it is possible to develop *in-vitro* release characteristics for 3D printed tablets beyond those possible for conventional tablets (3,4). Specific designs enabling tailor-made drug release behaviour include multilayer devices (3,5,6) or compartmental devices (5,7,8) comprising a different active pharmaceutical ingredient (API) in each layer or compartment. In the majority of applications developed to date, the 3D printing process is carried out by means of micro hot melt extrusion processes where molten polymer (or polymer/drug mixture) is deposited layer-by-layer to form a 3D object based on a computer aided design (CAD) in a process called fused deposition modelling (FDM). By careful design and selection of the filaments used for the extrusion it is possible to print coating barriers suitable for a range of immediate and modified-release applications (9).

However, while the structure and composition of the resulting dosage form can be designed in many new and innovative ways it is important to systematically challenge the applicability of existing quality control strategies for powder compacts as defined in the respective pharmacopoeias for ensuring the quality of 3D printed dosage forms. In a 3D printed tablet the (micro) structure of the dosage form is defined by design rather than being the result of the complex and hard to control properties of granular particulate mixtures. Furthermore, by definition batch release based on random sampling cannot be applied for patient-centred dosage forms in the traditional sense either. Although 3D printing has been a research topic over the last decades there remains a gap in understanding the impact of substrate materials, APIs, the method of incorporating the API into the 3D printed structure and the configuration of the printing process on the dosage form performance. Fundamental measurements and understanding of these interactions are essential to develop quantitative predictive models of the printing process and to guarantee a high product quality of every single dosage unit (10).

Given the unique ability to print extremely well defined structures, and the role these structures play in the design of the dosage form, it is clear that the microstructure will play a central role to define the drug release characteristics, and hence performance, for a 3D printed dosage form. In this context it is useful to highlight that in principle any thermoplastic pharmaceutical excipient can be utilised as a substrate material (filament), but that the print quality varies considerably depending on for example the melting point, thermal expansion coefficient, and elasticity of the filament as well as a range of process parameters such as filament feed rate, cooling rate and others (11). The impact of the structural accuracy and integrity of printed structures for different materials in the pharmaceutical context is relatively poorly understood. Sandler *et al.* (12) used scanning white light interferometry (SWLI) to determine the thickness and roughness of films of printed excipients and drug/excipient mixtures. They also used the same technique to rapidly determine the structure and presence of defects in printed drug delivery systems. Using this approach it was possible to separate layer structures with thicknesses as small as 0.5 μm in polymer films, but the method was not suitable to investigate samples with a thickness of more than a few millimetres. However, for 2D printed dosage forms SWLI proved a very powerful analytical technique.

One promising method to characterise 3D printed structures is X-ray computed microtomography (XμCT). The XμCT technique covers a range of spatial resolution, depending on sample size, of between 1-100 μm (13). Such a high spatial resolution can be achieved due to the short wavelength of X-rays and the availability of suitable detector arrays. Due to their high energy, X-rays have the advantage of being able to easily penetrate all pharmaceutically relevant excipients while exhibiting negligible diffraction (14).

Employing XμCT to characterise 3D printed structures has previously been performed in the field of scaffold-based tissue engineering for example for the examination of the mechanical deformation of 3D printed biodegradable poly(trimethylene carbonate) scaffolds (15) and the characterisation of the bone healing progress in calcium phosphate and collagen 3D printed scaffolds (16). The biological functionality of engineered tissue is highly influenced by architectural characteristics including porosity, pore size, surface area to volume ratio, interconnectivity, anisotropy, strut thickness (struts make up the interconnecting scaffold framework), cross sectional area and permeability (17,18). Most of these properties are of similar importance for 3D printed dosage forms (7,19). It was shown for 3D printed poly-ε-caprolactone (PCL) scaffolds that XμCT is perfectly suitable to analyse these characteristics like internal geometry, porosity and interconnectivity of pores (20).

XμCT enables the investigation of microstructures in great detail, but cannot be applied to control the microstructure of every single dosage unit due to its long acquisition and reconstruction time. An alternative to XμCT is terahertz pulsed imaging (TPI) allowing for the acquisition of single depth-resolved scans in a few milliseconds. TPI is a novel modality for physical characterization of pharmaceutical drug materials and solid dosage forms (21). Terahertz radiation easily penetrates through most polymeric materials (22) making it an attractive tool for non-destructive testing of pharmaceutical products. Applications for TPI include the direct measurement of coating thickness and the analysis of the uniformity of pharmaceutical film coated tablets, structural imaging and 3D chemical imaging of solid dosage forms (14,23). In TPI, the terahertz beam is focused onto the surface of the sample, where the main part of the radiation is directly reflected by the surface of the sample. A substantial fraction of the radiation penetrates into the structure and is then reflected back by subsequent interfaces separating two media with different refractive indices. Distances can be determined by measuring the delay time between the reflections of different structures and considering the refractive index of the material under investigation.

In this study we employed XμCT and TPI to qualitatively and quantitatively analyse the microstructure of 3D printed prototype solid dosage forms produced by FDM. Initially, the concept of the characterisation using XμCT and TPI is presented using the example of a simple hollow cylindrical shape dosage unit with one inner compartment prepared from two different polymer filaments: polyvinyl alcohol (PVA) and polylactic acid (PLA). The pore structure network is extracted from XμCT data and further analysed in terms of porosity, pore volume and pore length. In addition, the print resolution and quality is examined on the basis of the co-registered CAD model and the XμCT data of the dosage form. The same analysis was then applied on a multi-compartmental dosage unit filled with self-nanoemulsifying drug delivery system (SNEDDS) formulations containing API. The microstructural characteristics of the compartmental dosage forms were compared to their drug release profiles.

## Material and Methods

### Materials

**Fig. 1:**
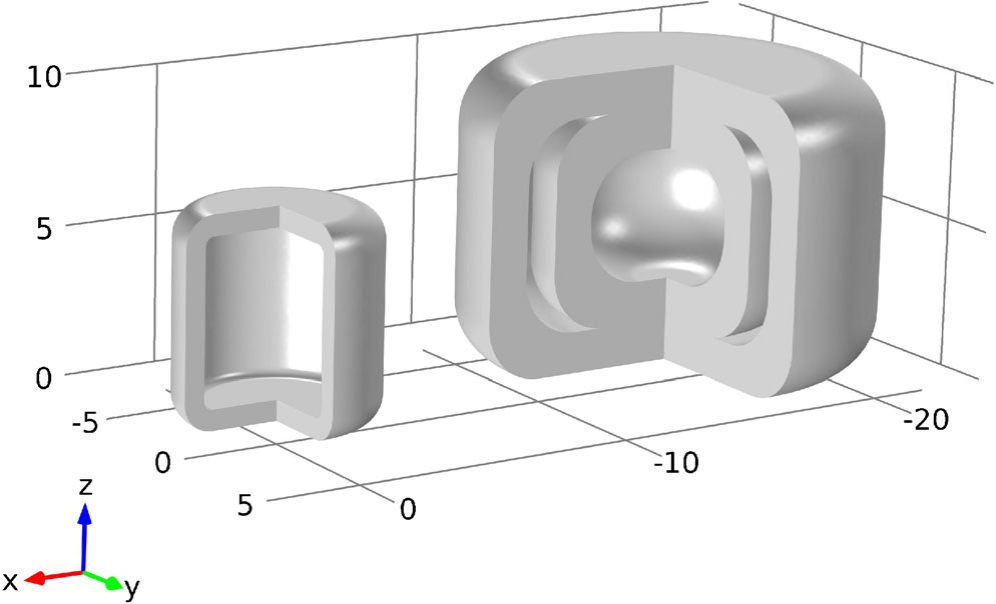
CAD schematics of cylindrical shape with one compartment (*left*) and with a two-compartmental design (*right*).

PVA and PLA from Innofil3D BV (Emmen, The Netherlands) were used as filaments for the printing of cylindrical structures with one or two compartments (see Fig. 1). Both filaments could be directly fed to the 3D printer. The average filament thickness was 1.765 ± 0.012 mm (*n* = 20) and 1.702 ± 0.004 mm (*n* = 20) for the PVA and PLA, respectively.

A complete list of all samples is provided in Table I. One sample of the one-compartmental dosage unit was filled with carbamazepine powder (CBZ, Hawkins, Inc., Minneapolis, MN, USA). The two-compartmental dosage units were filled with different liquids. The primary filling material was a SNEDD system consisting of soybean oil from Sigma-Aldrich (St. Louis, MO, US), Kolliphor® P 188 from BASF (Ludwigshafen, Germany), Maisine 35-1 from Gattefossé (Saint-Priest Cedex, France) and ethanol absolute from VWR international (Fontenay-Sous-Bois, France). SNEDDS containing saquinavir (0.05 g saquinavir / g SNEDDS) or halofantrine (0.05 g halofantrine / g SNEDDS) were used as filling material for the inner and outer compartment, respectively. The free base forms of saquinavir and halofantrine were both synthesized in-house from the hydrochloride salt. Other chemicals, such as organic solvents and buffering reagents were of analytical grade and obtained from Merck (Darmstadt, Germany) and Sigma-Aldrich (St. Louis, MO, US). The other filling material was silicone oil, which was used as a contrast agent for the XμCT measurements as it includes atoms of significantly higher electron density than the SNEDD system and thus more strongly absorbs X-rays (24).

**Table I:** Listing of all Samples Measured by XμCT

ID	Geometry	Shell material	Filling material
S01	One-compartment	PVA	Empty
S02	One-compartment	PLA	Empty
S03	One-compartment	PVA	CBZ
S04	Two-compartments	PVA	Empty
S05	Two-compartments	PVA	Empty
S06	Two-compartments	PVA	Empty
S07	Two-compartments	PVA	Silicon Oil (inner and outer compartment)
S08	Two-compartments	PVA	Silicon Oil (outer compartment)
S09	Two-compartments	PVA	Silicon Oil (inner compartment)
S10	Two-compartments	PVA	SNEDD system (inner and outer compartment)
S11	Two-compartments	PVA	SNEDD system (outer compartment)
S12	Two-compartmental	PVA	SNEDD system (inner compartment)

The samples S01 and S02 were also measured by TPI. The SNEDD system always contained saquinavir for the outer and halofantrine for the inner compartment. The sample ID is used throughout this study. PVA - polyvinyl alcohol; PLA - polylactic acid; CBZ – carbamazepine

### 3D Printing of Model Geometries

Cylindrical dosage forms with a single compartment (height: 7 mm, o.d.: 6.7 mm) and with two compartments (height: 10 mm, o.d.: 14 mm) were designed in Comsol Multiphysics (Comsol, Stockholm, Sweden, v5.1). The 3D CAD models are illustrated in Fig. 1. The nominal thickness of the shell is 0.7 mm for the one- and 1.4 mm (same thickness for the inner and outer shell) for the two-compartmental samples. In order to 3D print the geometries the Comsol CAD files were converted to binary STL (STereoLithography) files.

The samples were produced on a Makerbot Replicator 2 desktop 3D printer (New York, NY, US). This FDM printer uses a thermoplastic filament, which is heated to its melting point, extruded to produce a deposit strand with a width of 0.4 mm and a height of 0.3 mm. This deposit strand then creates a 3D object layer by layer. A MakerBot Replicator 2 running on MakerWare software (Makerbot, New York, NY, US, v 3.8.1) was configured with 100% infill and a 3D nozzle print temperature of 230°C. The printing process was stopped to enable the filling of the samples S03 with the CBZ and S07-S12 with the liquid formulations. After filling the printing process was restarted to close the samples. A video showing the 3D printing of a compartmental dosage form is available in the online supplementary material.

### Terahertz Pulsed Imaging (TPI)

The cylindrical shaped dosage forms were measured using a commercial TPI system (Imaga 2000, Teraview Ltd., Cambridge, U.K.). Five hundred and twelve data points were acquired for each terahertz time-domain waveform corresponding to a depth of 3.45 mm in air. Such a single terahertz time-domain waveform encodes information about the microstructure at one single point on the surface of the dosage form. In order to analyse the whole dosage form, it is necessary to point map across the entire surface of the sample. This is performed by an automated terahertz tablet scanner, which presents the dosage form at an angle of normal incidence to the terahertz optics in order to avoid distortions of the waveforms due to refraction of the terahertz beam on the dosage form surface. Therefore, the instrument generates a 3D dimensional model of the surface prior to the terahertz measurements and further uses this model to present any point on the surface of the sample at an angle of normal incidence to the terahertz optics. The terahertz mapping is thus performed for the top, bottom and side surface of the cylinder, whereas only the side surface is examined in this study. The side surface is described as a function of the azimuth angle (ψ) and the vertical position (*y*) in cylindrical coordinates.

The waveforms were deconvolved mathematically to highlight the structures and remove noise. The inverse filtering as employed in TPI includes a division of the sample waveform by the reference waveform in the frequency domain, which amplifies any high frequency noise in the signal. Therefore, the frequency domain division was coupled with a double Gaussian filter to suppress these noise (25). The signal processing of the waveforms was executed in Matlab (Mathworks Inc., Natick, Massachusetts, USA, vR2016a) and the deconvolved TPI data was visualised in Avizo Fire (FEI Company, Hillsboro, Oregon, USA, v8.1).

### X-ray Micro Computed Tomography (XμCT)

The 3D printed dosage forms were analysed using a SkyScan 1172 high-resolution XμCT scanner (Bruker, Antwerp, Belgium). The SkyScan 1172 utilises a cone beam geometry in combination with a 2D array detector. In this type of instrument the size of the sample and the resolution of the CCD array are the limiting factors for the maximum achievable spatial resolution given that shadow projects of the X-ray transmissions are recorded. Smaller samples can be magnified to a higher resolution. The samples were imaged at an isotropic voxel resolution of 2.97 μm and 5.00 μm for the one- (S01 – S03) and the two-compartmental samples (S04 – S12), respectively. 3D imaging is performed by rotating the object through 180° with steps of 0.25° and recoding the projection images (5 images were averaged per position) using the cone-beam configuration. A total of 720 images were thus generated during a total acquisition time of about 1.5 h per sample.

The subsequent reconstruction using NRecon (Bruker, v1.6.8.0) took about 30 min per sample. The data was downsampled during the reconstruction to a voxel resolution of 8.91 x 8.91 x 17.82 μm^3^ (924 x 924 x 405 pixels) and 14.99 x 14.99 x 28.98 μm^3^ (1060 x 1060 x 373 pixels) for the one- (S01 – S03) and the two-compartmental samples (S04 – S12), respectively. The downsampling was required to enable the processing of the data in Avizo Fire.

**Fig. 2:**
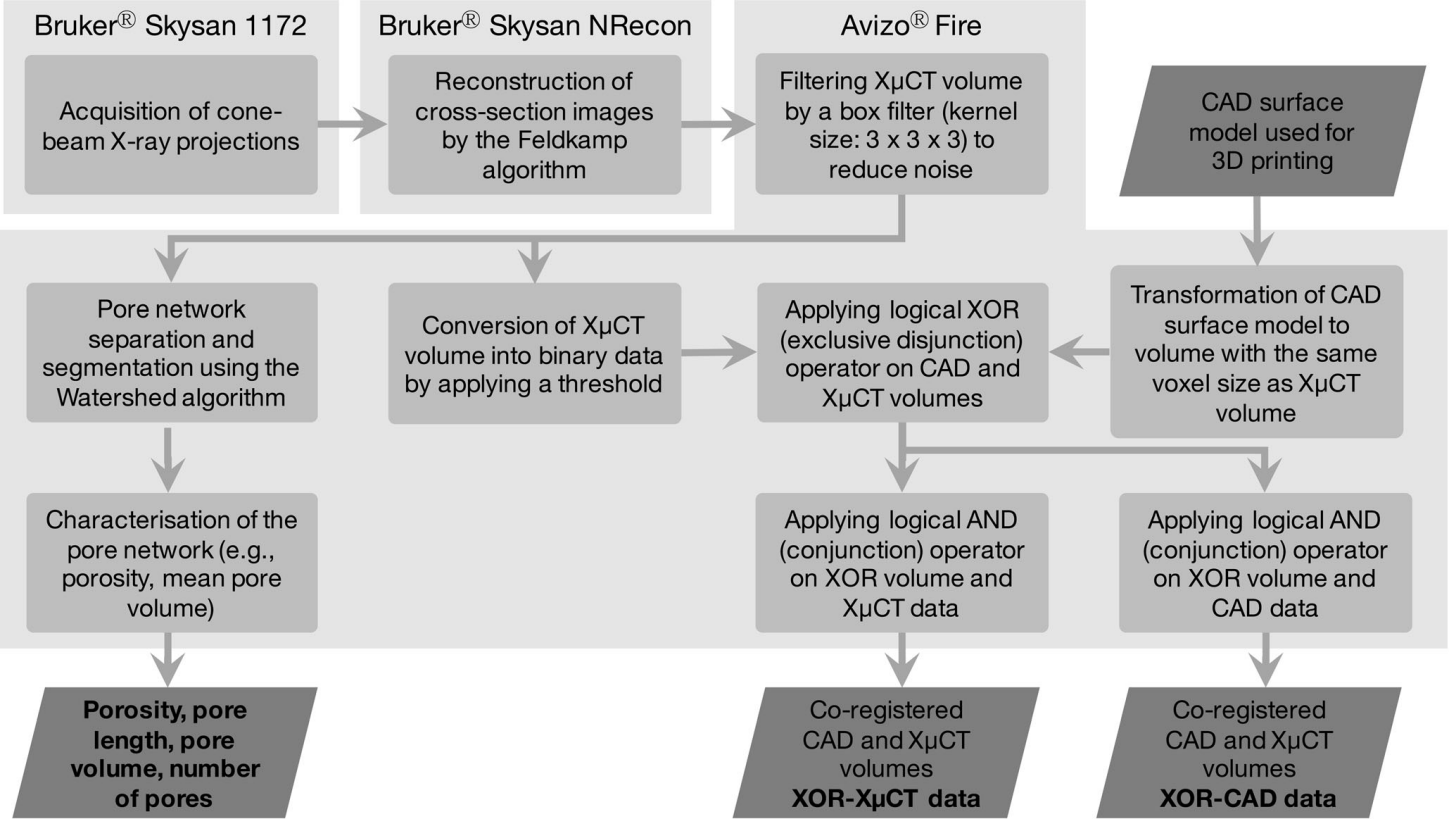
Overview of the XμCT data processing. Each *rectangular block* represents one single processing unit and the rhombus shaped blocks correspond to input or output data.

The schematic in Fig. 2 illustrates the basic data flow and used software for the acquisition and processing of the XμCT data. The processing consists of two main streams: (1) pore network characterisation and (2) co-registration of the XμCT and the CAD surface model of the 3D printed dosage forms. The core algorithm for the extraction of the pore structure is the watershed transform to separate touching objects in an image. It assumes the image gradient as a topographic map and finds catchment basins and watershed ridge lines. In order to improve the extraction of the pore network, we applied a marker-controlled watershed algorithm using defined foreground and background regions.

**Fig. 3:**
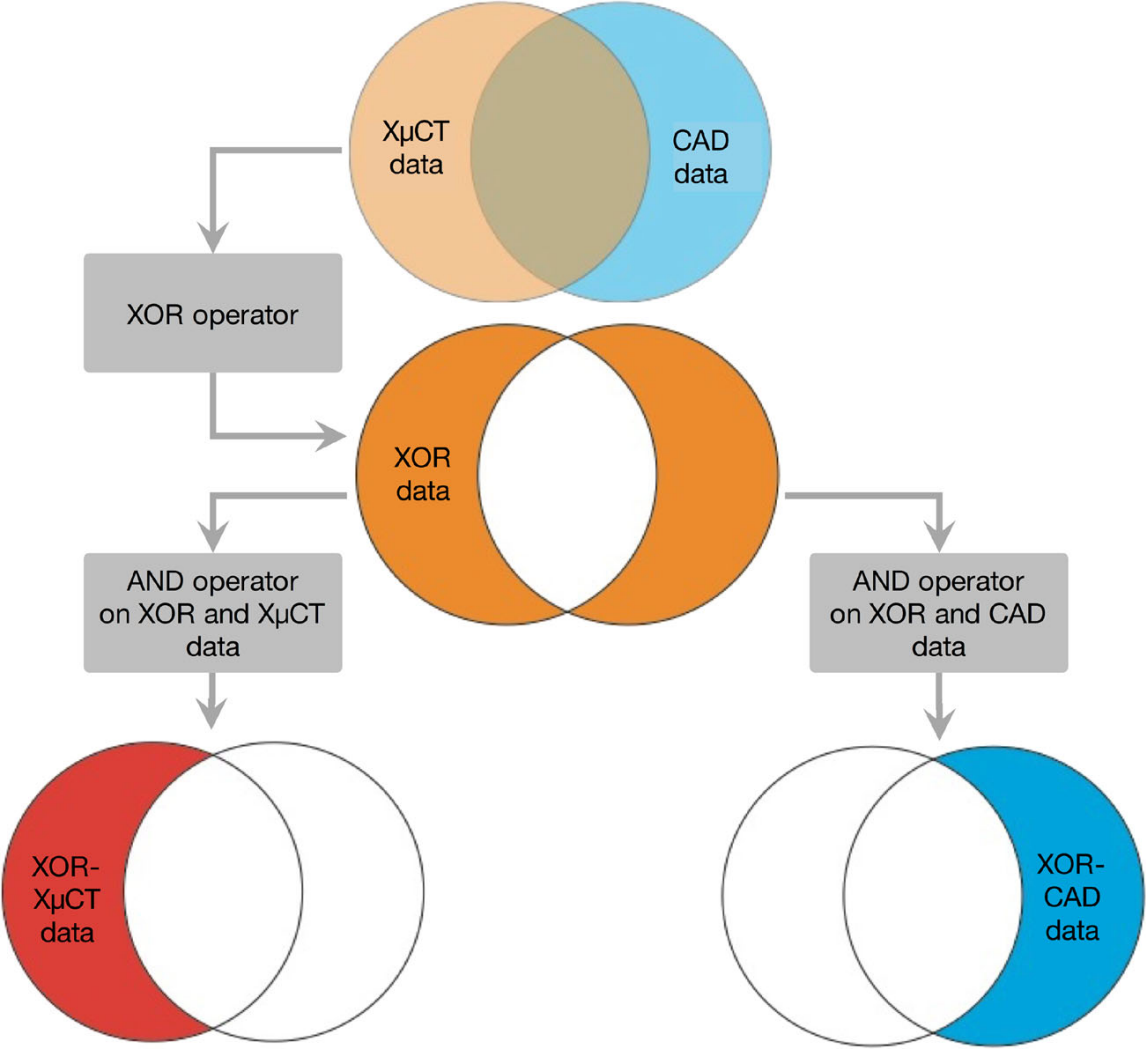
Schematic of the co-registration of XμCT and CAD data.

The co-registration of the XμCT and CAD data (same STL files as used for the printing) was conducted by applying logical operators as described in Fig. 3. The aim of this procedure is to evaluate the performance of the printing process. On the one hand, it identifies a subvolume of the XμCT data (henceforth referred to as XOR-XμCT data), which does not overlap with the CAD model. On the other hand, this approach is used to extract a subvolume of the CAD data (henceforth referred to as XOR-CAD data), which is not shared with the XμCT volume.

### Drug Release Testing

Drug release was experimentally determined by *in-vitro* release testing using the basket method (USP 1) in HCl pH 1 on a Erweka DT 70 (Heusanstamm, Germany) at 100 rpm and 37C and high-performance liquid chromatography (HPLC) analysis was carried out on a UHPLC+ Dionex Ultimate 3000 Thermo Fischer Scientific (Waltham, MA, US) as outlined previously (26). The drug release testing was performed for the two-compartmental samples with the SNEEDS formulation containing saquinavir in the outer and halofantrine in the inner compartment. The release profile is also compared to gelatine capsules filled with saquinavir.

## Results and Discussion

### Characterisation of 3D Printed One-Compartmental Geometries Using XμCT

**Fig. 4:**
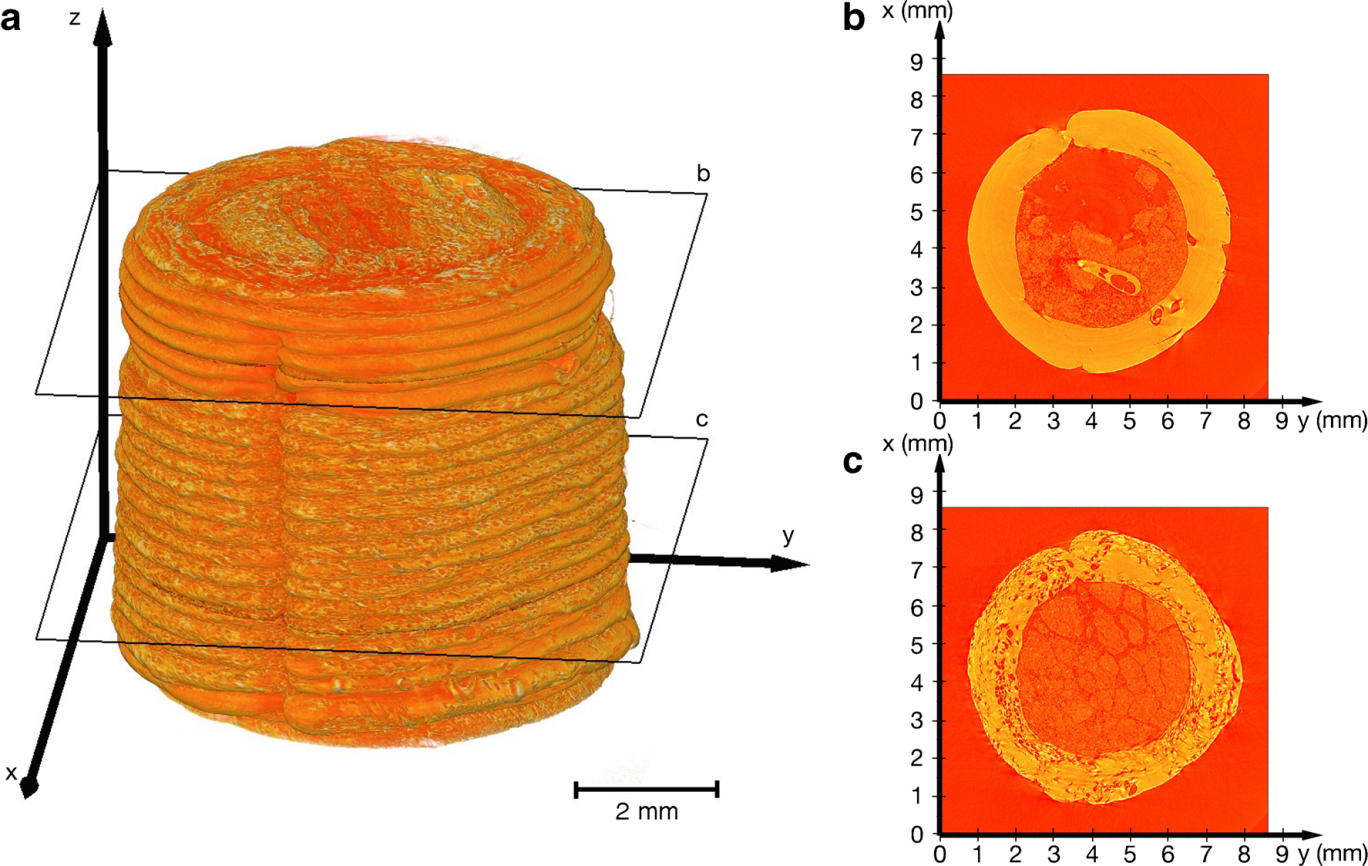
Visualisation of XμCT data of sample S03 (cylindrical PVA shell filled with CBZ). (**a**) 3D visualisation of XμCT data. (**b**, **c**) x-y cross-section images from the positions as denoted in (**a**).

Figure 4a shows the 3D rendering from the XμCT data of the 3D printed one-compartmental dosage form. The 3D rendering clearly illustrates the principle of FDM printing: the 3D object is created layer-by-layer from bottom to top; every single layer and the start of every flattened strand is noticeable in the XμCT data. This dosage form contained CBZ powder and thus the printing process was stopped to enable the filling. The stop of the process is visible in the 3D rendering (slightly below the cross-section label for Fig. 4b) as the diameter of the cylinder shrank from 6.97 ± 0.12 mm (*n* = 6) to 6.61 ± 0.05 mm (*n* = 6). Interesting differences in the internal structure of the polymer strands were observed: the cross-section image in Fig. 4b, corresponding to a section of polymer that was printed after the filling step, exhibits a relatively homogenous internal microstructure devoid or pores, which is in stark contrast to polymer structure that was printed before the filling step (see Fig. 4c). The change of the pore structure can also be observed in the 3D rendering by the rougher surface of the 3D print before the stop compared to the material printed after the filling. This indicates that this 3D printing platform needs some time to reach steady-state again and to produce a consistent structure within the entire dosage form. It can be observed towards the end of the printing run, i.e. at the top of the 3D rendering in Fig. 4a, that the porosity of the polymer strand is gradually increasing again. Figure 4b and c further visualise the CBZ particles inside the 3D print. The volume weighted mean particle size of the CBZ powder is about 12 μm and the structural domains that are visible in the cross-section images represent agglomerates of CBZ particles. In general, this type of analysis could be used to validate the internal fill volume as well as to evaluate particle agglomeration.

**Fig. 5:**
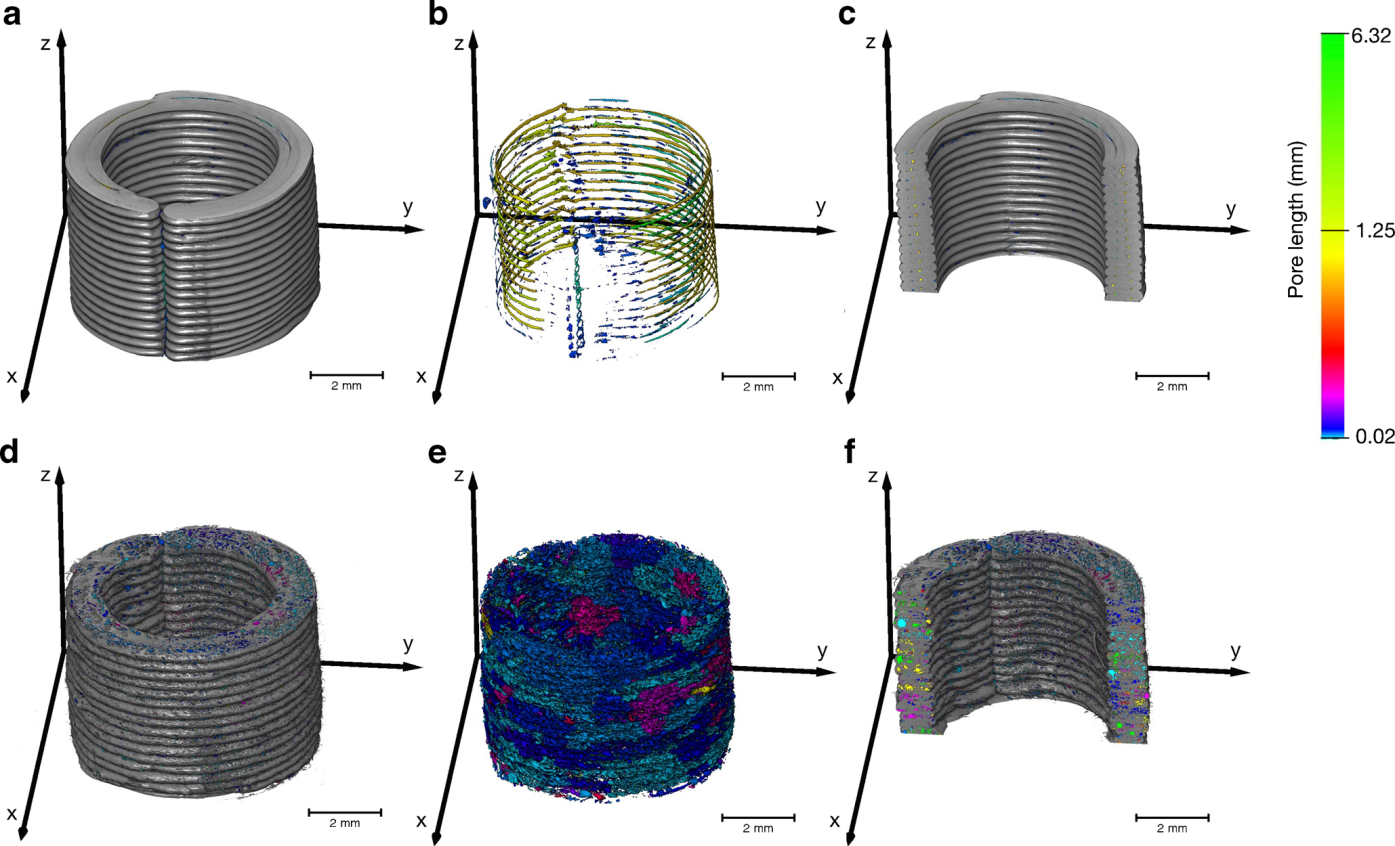
Analysis of pore structure of (**a**,**b**,**c**) PLA and (**d**,**e**,**f**) PVA shells on the basis of XμCT data. (**b**) and (**e**) illustrates only the pores, where a colour depending on the pore length was assigned to each connected pore. (**c**) and (**f**) are y-z cross-section images of the PLA and PVA shell, respectively. The colour map is valid for all subfigures.

A detailed investigation of the pore structure within the wall polymer strands was conducted for the empty PLA and PVA samples. In these samples the pore structure is consistent over the entire 3D printed structure as there was no filling step and the process was not stopped. Figure 5 highlights a clear difference between the PLA and PVA samples in terms of number of pores and pore lengths. In particular, long tube-like pore structures form between each layer when PLA is used as a filament. The deposit strand width is 0.4 mm (the hardware parameter of the printer is set by the extrusion nozzle diameter) and therefore the printer uses two strands to build each layer of the wall (target thickness 0.7 mm). The tube-like pores are located between the two neighbouring strands and between each successive layer. Such pores are not present in the print when PVA is used as the filament. However, the use of PVA results in a more complex pore structure network of much higher porosity formed by smaller pores (Table II). The pores in the PVA samples exhibit high connectivity and hence appear as clusters in Fig. 5e. However, the Watershed algorithm also separates a high number of small pores from the connected pores leading to a high standard deviation (SD) of the mean pore volume for the PVA filament. Using these data the total porosity can be determined (fraction of void volume to total volume) yielding 5.5% and 0.2% for the PVA and PLA samples, respectively. The quantities in Table II highlight the significant difference in terms of the internal microstructure between the two filament materials. In addition, Fig. 5a and b further reveal that there are voids between the start and the end of each strand in each layer indicating a systematic defect of the dosage unit, which might act as a weak spot to containment of any filling and where dissolution medium might be able to penetrate more quickly into the dosage form. These voids are more pronounced in PLA than in PVA samples.

**Table II:** Characteristic Microstructural Properties of the Empty One-Compartmental PVA (Sample S01) and PLA Shells (Sample S02)

		S01	S02
Weight	mg	145.2	161.3
Porosity	%	5.5	0.2
Mean pore volume	mm^3^× 10^4^	2.70 ± 141.80	3.80 ± 4.00
Mean pore length	mm	0.04 ± 0.09	0.20 ± 0.72
Shell thickness (*n* = 6)	mm	1.11 ± 0.05	0.99 ± 0.10
Deposit layer thickness	mm	0.27 ± 0.03	0.28 ± 0.01
XOR-CAD volume	mm^3^	38.6	15.1
XOR-XμCT volume	mm^3^	32.7	33.9
Total XμCT volume	mm^3^	108.6	129.1
XOR-CAD volume / CAD volume	%	33.7	13.7
XOR-XμCT volume / CAD volume	%	28.5	30.7
XμCT volume / CAD volume	%	94.8	117.1

The total CAD volume is 115.07 mm^3^. The total volume of the XμCT in the table is the volume without the void spaces. The difference between the XOR-XμCT volume / CAD volume and XOR-CAD volume / CAD ratios is approximately the same as XμCT volume / CAD volume – 100. The deposit layer thickness is the vertical layer height, which should be nominally the same as the deposit strand height of 0.3 mm

**Fig. 6:**
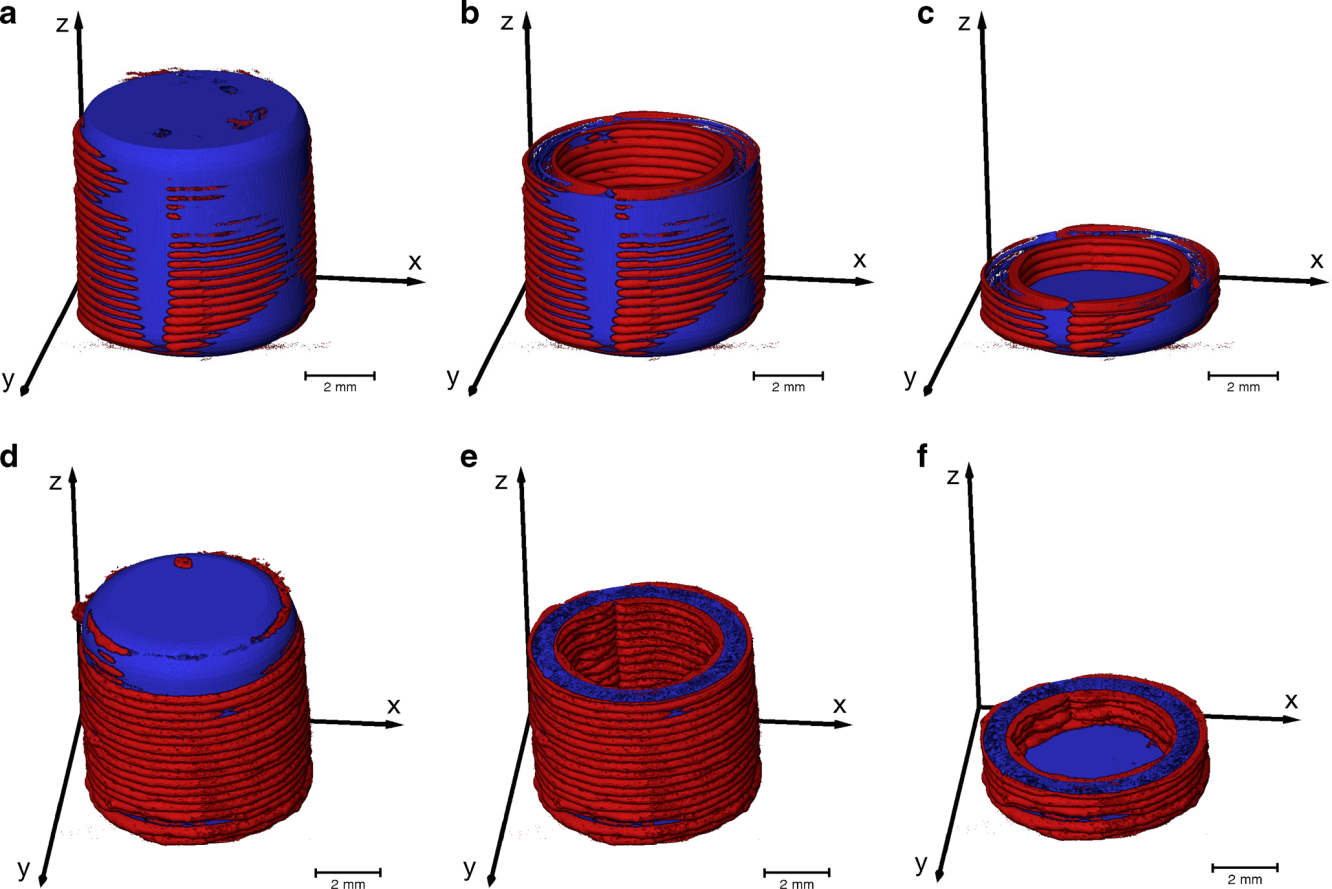
Co-registration of the 3D rendering of the XμCT images and the CAD model. The *blue color* visualises the XOR-CAD data and the *red color* represents the XOR-XμCT data. (**a**,**b**,**c**) are 3D printed using PLA and (**d**,**e**,**f**) represent the XμCT results of the PVA shells. The online supplementary material contains two videos of the co-registered XμCT data and CAD model of the samples S01 and S02

The volumes of the XOR-XμCT (red) and XOR-CAD (blue) data, as given in Table II, were computed from the co-registered images (Fig. 6). The XOR-XμCT volume indicates the excess of material and the XOR-CAD volume reveals the material that is missing in the 3D printed structure. The aim of this analysis is to quantify the printing accuracy relative to the ideal CAD model, which was utilised to guide the printing process, and can be further applied to optimise the printing. The volume of the 3D printed PVA sample is smaller than it was designed to be due to the large pore volume, although it appears larger in Fig. 6. The PVA shell is about 59% thicker than designed, whereas the PLA shell thickness increases in average by 41%. Moreover, the deficiency ratio (XOR-CAD volume / CAD volume) is significantly lower for the PLA sample due to the smaller pore volume and this also leads to a larger ratio between the total XμCT and the CAD volume.

### Microstructural Characterisation of the One-Compartmental Samples Using TPI

One of the key challenges in patient-centred medicine is the quality control of each single dosage unit. Although XμCT provides very detailed information about the microstructure used to identify defects in the 3D print, it is unfeasible to control every single printed dosage form due to its long acquisition and reconstruction times (>1 hour). TPI could act as an alternative quality control tool providing fast acquisition of depth profiles (<1 s) and thus enabling the control of a much higher number of samples. However, the interpretation of the terahertz waveforms is more complex and prior knowledge based on the XμCT measurements has to be developed in order to relate the TPI data to microstructural properties relevant for quality control.

**Fig. 7:**
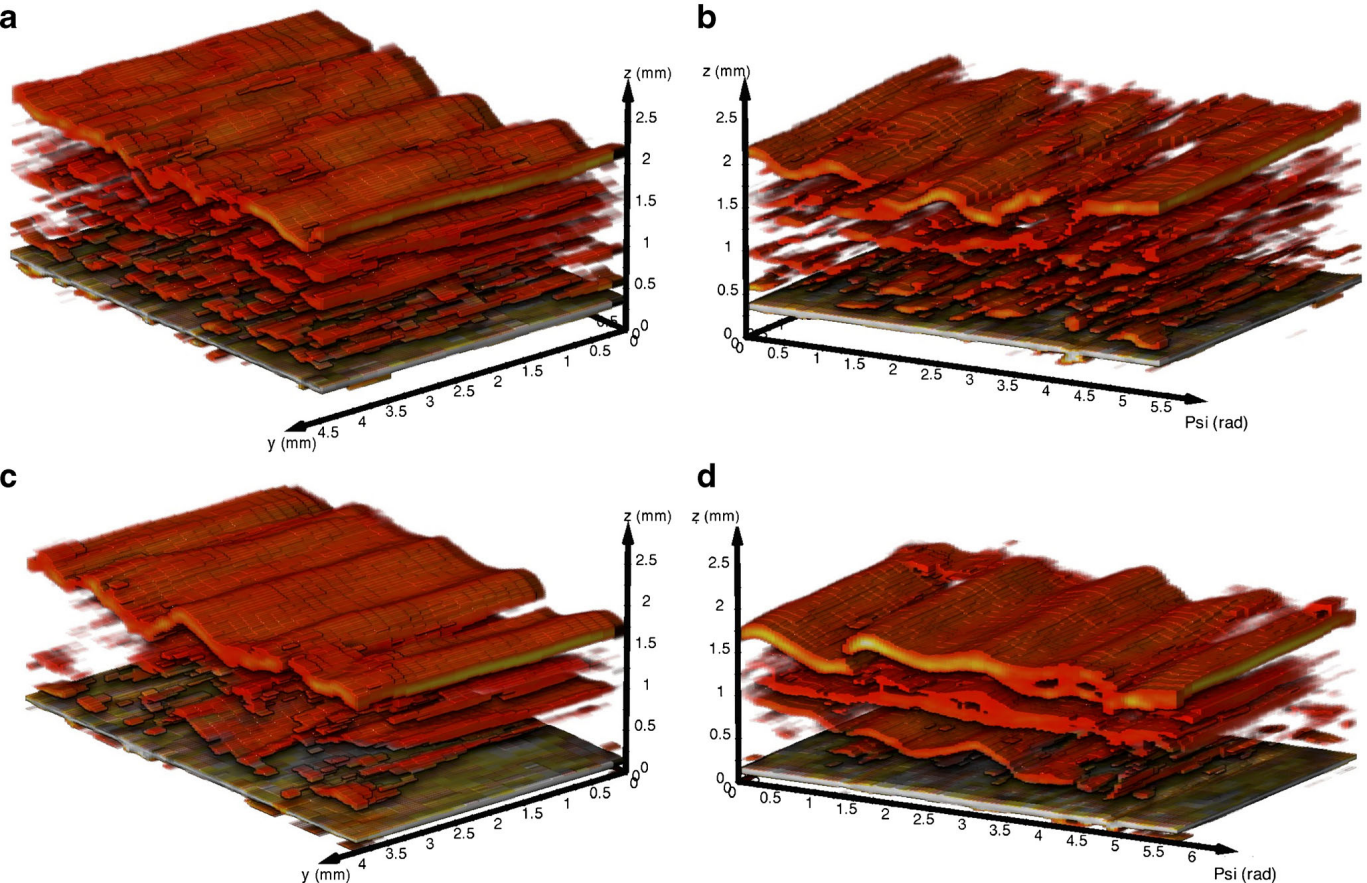
3D TPI data of (**a**,**b**) empty PVA shells (sample S01) and (**c**,**d**) empty PLA shells (sample S02). The bottom layer in each 3D image corresponds to the air/shell interface. Coordinate system: Psi is the azimuth angle in accordance to a cylindrical coordinate system; y corresponds to the vertical position on the cylindrical shaped sample; z is the depth coordinate considering a refractive index of 1

3D renderings from the TPI data (Fig. 7) indicate differences between microstructure of the PVA and PLA samples. In accordance to the XμCT measurements, a more complex network of pores is visible in the PVA (Fig. 7a and b) than in the PLA (Fig. 7c and d) samples. The supplementary information additionally presents peak intensity maps of two samples, which clearly highlight defects on the surface (i.e., low peak intensity) of the dosage forms. The peak intensity is strongly affected by the refractive index of the surface and can thus be used to analyse relative density changes. The terahertz measurements therefore provide additional information about the quality of the dosage forms, as such strong variations of the surface were not observed in the XμCT data. Furthermore, terahertz imaging allows to control the shell thickness, which directly impacts the drug release kinetics, in a non-destructive and contactless manner. The measured shell thickness values are 1.12 ± 0.05 mm (*n* = 6) for the PVA (refractive index of 1.6 (27)) and 0.86 ± 0.02 mm (*n* = 6) for the PLA (refractive index of 1.89 (28)) shells, which are in good agreement with the thickness measurements from XμCT (see Table II).

The quantitative interpretation of the terahertz data is not straightforward as the terahertz pulses are focused to a diffraction limited spot of 200 μm diameter to the surface of the printed dosage form. This configuration is specifically designed to investigate relatively thin subsurface structures such as film coating layers that extend to a depth of several hundred micrometres in *z*-direction at most. Given the penetrative power of terahertz radiation into the polymer materials used to print the structures it is possible to extract further structural information at depth from the data. The results clearly show that the inside wall of the printed structure can be resolved comfortably at depth > 1.5 mm. However, due to the increasing dispersion of the focused pulses at depth as well as the relatively strong scattering given the size of the pore structure it would be premature to draw full conclusions on the applicability of TPI for quantitative porosity analysis in such dosage forms. There is clearly a significant potential of this technique for such applications, which remains to be explored and which might require adjustments both to the terahertz optics, as well as the signal processing and data extraction routines that go beyond the remit of this proof-of-principle study.

### Characterization of Empty 3D Printed Two-Compartmental Geometries

Consequently, the internal structure of the more complex 3D printed two-compartmental geometries was only assessed by XμCT. The same analysis procedure as outlined in the previous section was conducted for the two-compartmental samples and the results are summarised in Table III. For these samples the actual sample volumes were found to be 7.5% ± 0.75 SD (*n* = 3) larger than the design. It is interesting to note that the volume of the cylindrical PVA samples was significantly smaller compared to the CAD volume, even though the porosity is similar in all cases. The different geometries might impact on the total volume as indicated by the deficiency (i.e. XOR-CAD/CAD volume) and excess ratios (i.e. XOR-XμCT/CAD volume) and we note that the difference between the two ratios is smaller, even negative, for the cylindrical compared to the compartmental samples. Furthermore, both the inner and outer shell thicknesses are considerably above the nominal value of 1.4 mm (36%) resulting in more excess material. This is in good agreement with several other studies (16,29) indicating a systematical deviation of the 3D print from the CAD model.

**Table III:** Characteristic Microstructural Properties of the Empty Compartmental Samples (S04-S06)

		S04	S05	S06
Weight	g	1.3119	1.3230	1.2959
Porosity	%	4.77	4.85	4.89
Mean pore volume	mm^3^ × 10^4^	6.10 ± 355.00	6.29 ± 40.00	4.96 ± 266.66
Mean pore length	mm^3^	0.09 ± 0.19	0.08 ± 0.18	0.09 ± 0.16
Outer shell thickness (*n* = 6)	mm	1.90 ± 0.08	1.92 ± 0.10	1.86 ± 0.09
Inner shell thickness (*n* = 6)	mm	1.96 ± 0.09	1.92 ± 0.08	1.90 ± 0.08
Deposit layer thickness	mm	0.29 ± 0.01	0.29 ± 0.01	0.28 ± 0.01
XOR-CAD volume	mm^3^	167.18	161.86	176.13
XOR-XμCT volume	mm^3^	233.05	245.13	273.58
Total XμCT volume	mm^3^	1199.54	1199.92	1214.16
XOR-XμCT / CAD volume ratio	%	20.80	21.88	24.42
XOR-CAD / CAD volume ratio	%	14.92	14.45	15.72
XμCT / CAD volume	%	107.08	107.12	108.39

The total CAD volume is 1220.2 mm^3^. The same relationships between the different quantities apply as described in Table II. The online supplementary material contains a video of the co-registered XμCT data and CAD model used to calculate the XOR-CAD and XOR-XμCT volumes of sample S04

**Table IV:** Properties calculated from the XμCT and CAD data for samples S08 (compartmental sample filled with silicon oil in the outer compartment) and S11 (compartmental sample filled with SNEDDS containing saquinavir in the outer compartment)

		S08	S11
XOR-CAD volume	mm^3^	121.8	153.1
XOR-XμCT Volume	mm^3^	266.1	276.1
Total XμCT volume	mm^3^	1261.0	1239.7
XOR-XμCT volume / total CAD volume	%	23.8	24.7
XOR-CAD volume / total CAD volume	%	10.9	13.7
XμCT volume / CAD volume	%	112.6	111.0
Volume of fill material	mm^3^	56.4	35.1

The volume of the fill material is calculated by subtracting the average total XμCT volume of 1204.54 mm^3^ of samples S04 – S06 from the total XμCT volume of the respective sample containing the liquid

As outlined previously, the printer with the configuration used for this study can only produce shell thicknesses with a discrete step size of 0.4 mm, which is limited by the physical width of the deposited printed strand. The 2-compartmental shells were produced by four adjoined strands yielding a wall thickness of 1.6 mm for each shell. The fact that the layer thickness in *z*-direction (see Table III) is slightly smaller (0.29 mm) than the nominal value of 0.3 mm (which was the chosen layer height) indicates that the strand deforms under its own weight causing an increase of the wall thickness in horizontal dimension by 13.3% (4(0.30–0.29)/0.30) for sample S04 using four strands in one layer) due to its contraction in vertical direction. This would yield a shell thickness of 1.90 mm (1.6 mm (1 + 0.133 + 0.0477) considering the measured porosity of 4.77% (for sample S04), which is in good agreement with the measured shell thickness. Based on the understanding of the dimensional changes due to gravity and porosity extracted from the results the CAD file could be modified to account for the volumetric changes of the material in order to produce more accurate prints. A modification of the process to achieve the desired dimensions of the 3D print could be performed by developing a predictive model which enables the selection of a suitable manufacturing procedure (including the design of the CAD model and process parameters), as proposed by Boschetto and Bettini (30).

However, the variation between repeated prints of the same design is very small and the microstructural parameters as listed in Table III are overall in very good agreement between samples. The small shell thickness variations might originate from the thickness variation of the PVA filament itself (9), which has an average thickness of 1.765 ± 0.012 mm (*n* = 20). Our results show that this printing technology provides a high reproducibility of the output for repeated prints of the same design. At the same time the results highlight that the accuracy of the production of a specific CAD model highly depends on the material properties of the feed materials and the design of the printed geometry. In particular, a thorough understanding of the rheology of the filament is necessary to produce high quality prints (31).

### Characterization of Filled 3D Printed Two-Compartmental Geometries

**Fig. 8:**
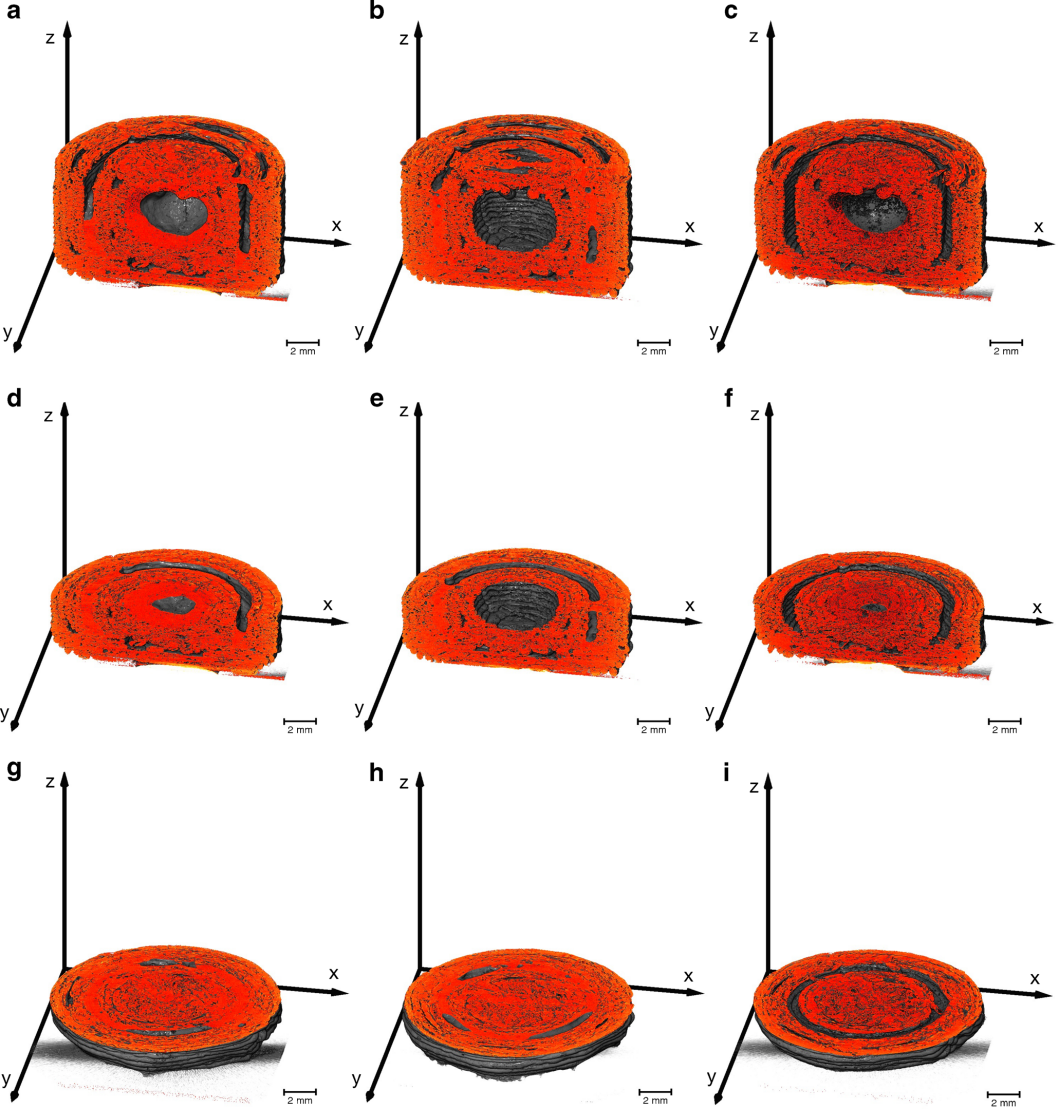
Subvolumes of the two-compartmental geometries filled with a SNEDD system generated from the XμCT data. The brighter regions correspond to the SNEDDS, which are noticeable in the outer and inner compartment (sample S10) in (**a**), (**d**) and (**g**), only in the outer compartment (sample S11) in (**b**), (**e**) and (**h**), and only in the inner compartment (sample S12) in (**c**), (**f**) and (**i**).

**Fig. 9:**
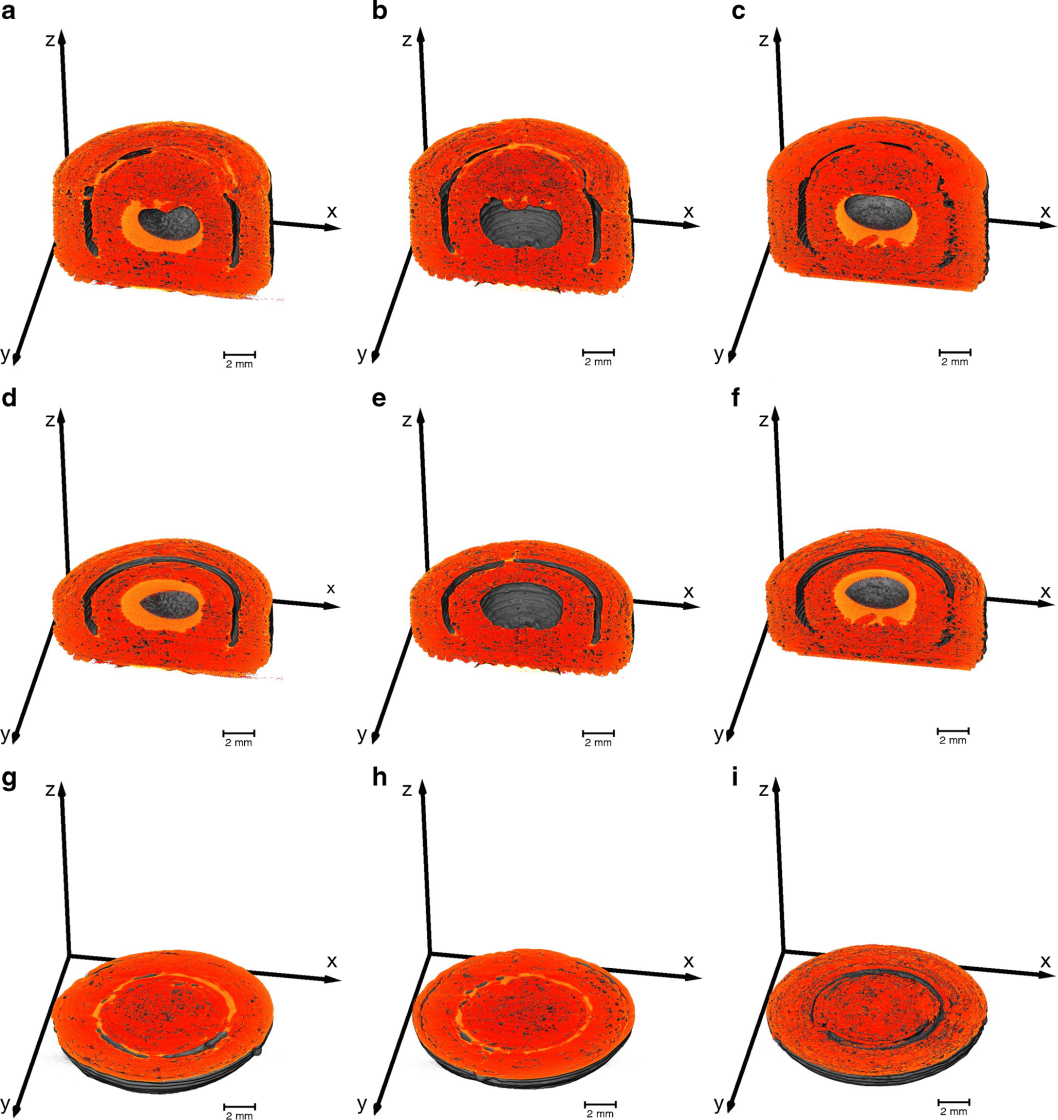
Subvolumes of the two-compartmental geometries filled with silicon oil generated from the XμCT data. The brighter regions correspond to the silicon oil, which is visible in the outer and inner compartment (sample S07) in (**a**), (**d**) and (**g**), only in the outer compartment (sample S08) in (**b**), (**e**) and (**h**), and only in the inner compartment (sample S09) in (**c**), (**f**) and (**i**). The online supplementary material shows a video of sample S07

The 3D printed two-compartmental structures were filled with silicon oil or with SNEDDS containing an API. The XμCT rendering of these samples are visualised in Fig. 8. The results show that the filling material cannot be easily differentiated from the polymer, in particular for the case where the liquid is loaded into the outer shell. However, areas with a different contrast are visible in Fig. 8h, which is assumed to be the SNEDDS (samples without filling did not contain this contrast, data not shown). In order to test this hypothesis we examined samples filled with silicon oil in order to artificially enhance the contrast in the X-ray shadow images. The presence of Si in silicon oil provides a significant difference in electron density compared to the SNEDDS and the polymer and thus makes it easier to distinguish the liquid from the PVA structures. This is illustrated in Fig. 9 where the silicon oil can be clearly distinguished from the PVA. The images show that the liquid is distributed in a similar fashion within the inner and outer compartment as the SNEDDS depicted in Fig. 8, albeit with higher contrast.

The XμCT renderings of filled samples indicate that the liquid exhibits a high attraction to the shell walls. In the inner compartment the fluid covers the entire surface and does not fill the open space from bottom to top as one might expect. A capillary pressure induced by the porous system might exert a force on the fluid, which facilitates penetration into the porous system. The surface tension of the oil then further causes the formation of an air bubble in the centre of the void which in turn results in the full coverage of the PVA shell surface. However, due to the high viscosity of the liquid the capillary pressure is too small to exert a sufficient force on the fluid that would result in deeper penetration of the oil phase into the porous structure or even an efflux of the fluid. A similar phenomenon can be observed Fig. 9b, e and h when the oil is placed into the outer compartment: the fluid is attracted to the shell surfaces that exhibit the largest curvature. This allows for the fluid to acquire the least surface area possible and yields menisci that cover the top and the bottom internal surface within the outer compartment.

**Fig. 10:**
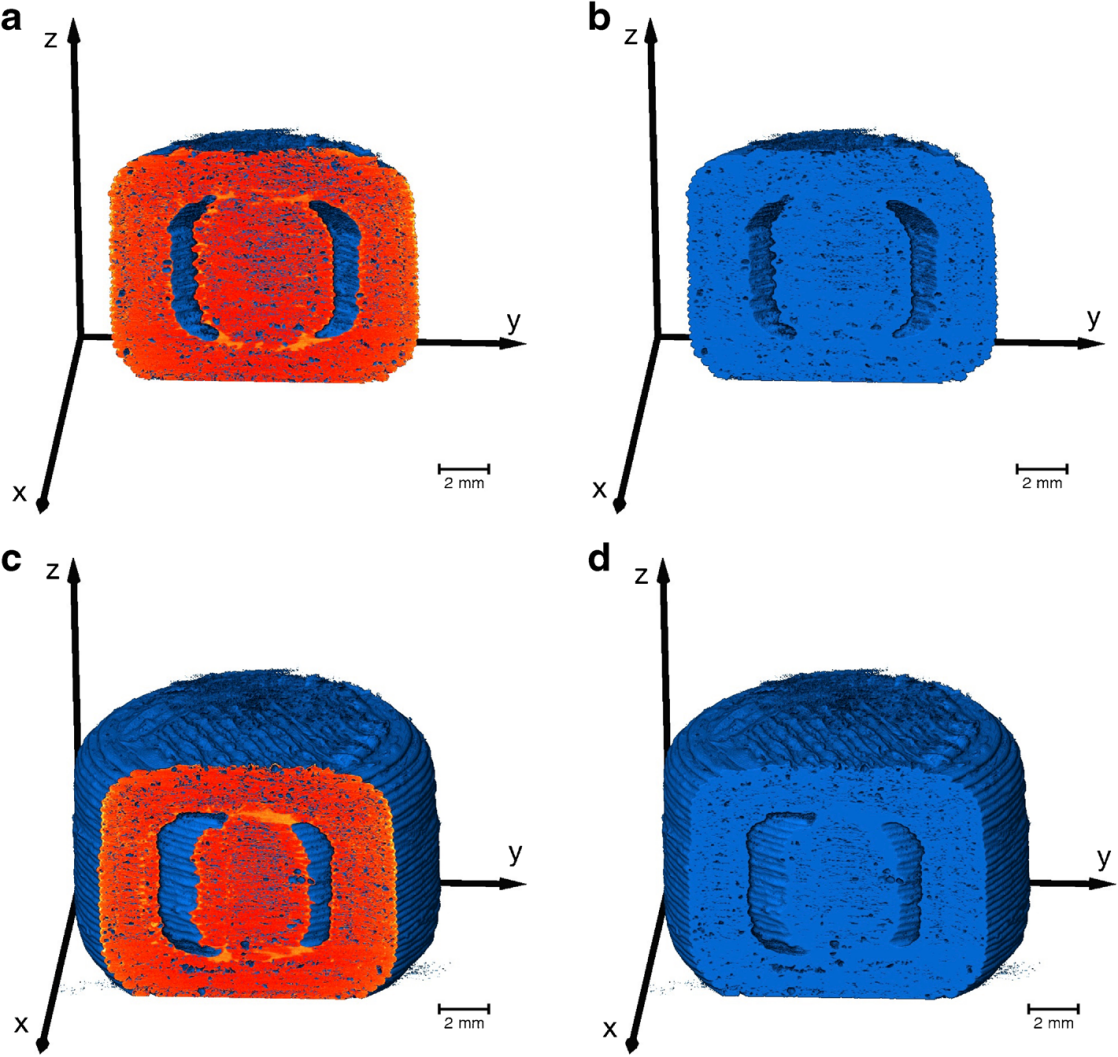
3D renderings of two different subvolumes of the same sample S08 (compartmental sample filled with silicon oil in the outer compartment). The volumes are binary data (0 – air, 1 - material) converted from the XμCT data by setting a threshold. Both pairs (**a**,**b**) and (**c**,**d**) present the same subvolume, where (**a**,**c**) additionally depicts the *y-z* cross-section from the original XμCT volume. The bright regions in the cross-section images (**a**,**c**) correspond to the silicon oil, which are also part of the binary data as depicted in (**b**,**c**). The liquid volume is thus included in the calculation of the material volume of the binary data.

In order to calculate the liquid volume, we converted the XμCT volume to binary data (Fig. 10). The threshold required for the conversion of the XμCT volumes to binary data was set on the basis of including the shell material and the silicon oil in the converted material. Slight variations of the threshold were not critical as the silicon oil could be readily observed in the *y-z* cross-sections (i.e. the bright regions) in Fig. 10a and c. Upon comparison of Fig. 10a, c with b, d it is clear that the converted binary data covers the PVA shell and the silicon oil. Therefore, the volume of the liquid in each compartment can be determined by subtracting the average volume of the empty structures from the total XμCT volumes (see Table IV for S08 and S11). Such analysis results in a volume of 56 mm^3^ and 58 mm^3^ for the case of the silicon oil in samples S08 and S09, respectively and, more importantly, a volume for the SNEDD system of 35 mm^3^ and 54 mm^3^ for samples S11 and S12, respectively. The liquid volume of the samples filled in both compartments are 101 mm^3^ (S07, silicon oil) and 97 mm^3^ (S10, SNEEDS). The current processing procedure provides only a total liquid volume and cannot differentiate between the volume from the inner and outer compartment. The separation of the liquid from different compartments as well as from the polymer will be implemented for future studies, which will also improve the accuracy of the volume measurement. However, these results are in surprisingly good agreement with the actual loading amount of 50 μl of liquid in each compartment, especially when taking into consideration the limited resolution of the used XμCT setup, the use of an average volume of the empty structure as well as the low contrast between SNEEDS and the polymer. Based on these results we conclude that XμCT can be used to measure the volume of loaded SNEDDS within the compartmental dosage forms. The analysis further validates that the liquid stays within the dosage form and does not diffuse significantly through the porous shell.

### API Release of Compartmental Dosage Unit

**Fig. 11:**
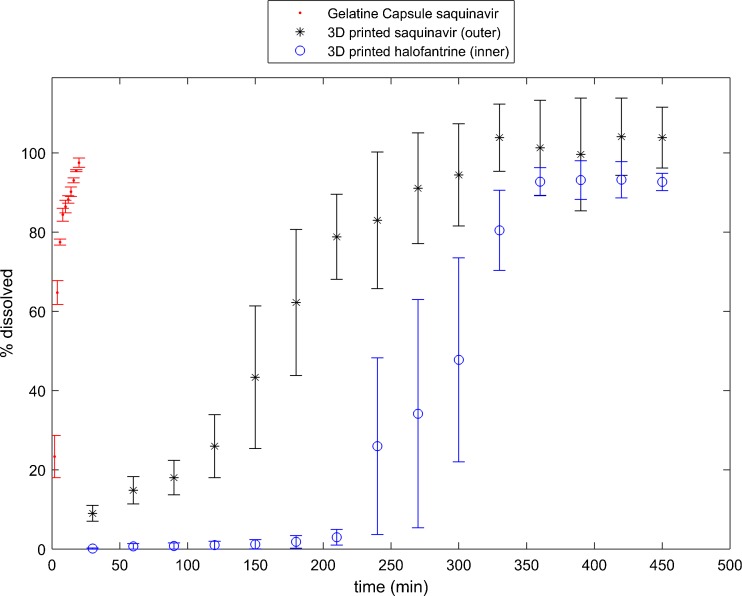
Drug release of APIs from gelatine capsules and 3D printed compartmental geometry filled with SNEDDS containing saquinavir in the outer compartment and halofantrine in the inner compartment. (*n* = 3, mean ± standard error of the mean).

The amount and combination of the SNEDDS containing APIs depends on the patients’ drug combinatorial needs and dose requirements. The release behaviour from the SNEDDS loaded two-compartmental printed dosage unit is contrasted to the release from conventional gelatine capsules. During dissolution testing it was observed that the two-compartment 3D printed dosage forms exhibited a considerable delay in release of API compared to release of API from gelatine capsules. As expected, the release of halofantrine from the inner compartment commenced only after approximately 240 minutes where roughly 80% of the saquinavir from the outer compartment was already released (Fig. 11).

The drug release characteristics are directly impacted by many factors such as the microstructural properties of the PVA shells. The mass transport of the dissolution medium and the drug might be driven by a pressure gradient (typically referred to as Darcy flow (32)), by an activity gradient (case II relaxation) or by a combination of both. Pressure and activity gradient are considerably impacted by microstructural properties of the shell pore structure (33). The total flux is highly affected by the properties of the porous structure (e.g., shell thickness, permeability, diffusivity, porosity, tortuosity and connectivity of the pores), the properties of the fluid (e.g., pH, viscosity, temperature) and properties of the fluid/porous system (e.g., contact angle/wettability) (34). The release of halofantrine from the inner compartment will be further delayed (210 min) as the dissolution medium and the drug have to pass two shells. Furthermore, the inner compartment exhibits a smaller surface area compared to the outer compartment which further reduces the API flux.

Moreover, the XμCT analysis indicates a strong wetting behaviour of the liquid formulation and the polymer wall leading to a significant adhesive force between the two materials. The dissolution medium and the lipid formulation have a far less attractive relationship. These surface interactions of the liquid formulation influence the release rates of saquinavir and halofantrine: In the outer compartment there are two surfaces for the liquid formulation to interact with while there is only one in the inner compartment and the inner compartment has as smaller surface area than the outer compartment.

The wettability also influences the forces (capillary and diffusion), which cause the penetration of the dissolution medium into the dosage form and the release of the liquid formulation out of the dosage form. The low release rate at the beginning (<150 min for the outer compartment in Fig. 11) indicates that the droplet diffusion of the SNEDD system is a slow process. During the experiment, erosion of the wall will also contribute to the diffusion (35). Erosion will change the pore structure in the shell by increasing the porosity with increasing dissolution time. This will further affect the interconnectivity of the pores and eventually facilitate the formation of micro-channels and late on actual channels. A capillary force builds up in these micro-channels causing a faster release process (>150 min for outer compartment in Fig. 11).

The different release kinetics between the two compartments can also be analysed by fitting a power law *y* = *kt* ^*m*^ (with y as the dissolved API% and *t* as the dissolution time) to the release data. This yields the fit parameters m = 0.80 and k = 0.88 for saquinavir and m = 0.63 and k = 3.29 for halofantrine. We only considered the release data of halofantrine above 210 min to reflect the release kinetics without the delay. The difference in exponent *m* indicates that the fundamental release processes are distinctly different, where the release of halofantrine is closer to a $\sqrt{t}$ -dependent release process. The large difference in *k* is mainly due to erosion of the inner shell during the release of the liquid from the outer shell.

The mass transport mechanisms involved in the disintegration and erosion process might be different for the same materials when 3D printed than when compressed to a tablet. During compaction tablet constituents are subject to plastic and elastic deformation before interparticulate bonds are forged (36). The reversible viscoelastic process of deformation, i.e. strain recovery, is one of the key mechanisms involved in the disintegration and erosion process of a tablet in the GI tract (37,38). Since there is no deformation of particles during 3D printing, strain recovery might not have any significant effect on the release behaviour of 3D printed solid dosage forms. Therefore, drug release behaviours of specific formulations cannot be directly translated to 3D printed products, even for dosage forms based on the same formulation. More research has to be conducted to better understand the drug release mechanisms involved in 3D printed dosage forms. A deeper understanding of the drug release mechanisms and the possibility to easily design product geometries are key elements for the future product design with increasing implementation of *in silico* principles (39).

## Conclusion

This study demonstrated the capability of using XμCT to develop a knowledge base for 3D printed solid dosage forms. A full exploitation of FDM and the relevant pharmaceutical applications requires a detailed examination of the printed microstructure in order to eventually manufacture consistent and high quality products. XμCT is highly suitable to characterise the architecture of the 3D print as well as to verify the print resolution, print quality and to confirm the added drug volume amount. The ease, speed and limited safety concerns of TPI compared to XμCT renders it a feasible platform for quality control of 3D printed patient-centred medicines in future. However, more research has to be performed to extract the essential information about the microstructure from the terahertz waveforms, which is limited at present.

3D printed compartmentalised formulations allow tailoring the *in-vitro* release profile of the respective drugs in the SNEDD system to target different parts of the GI tract. In particular, the microstructural information extracted by XμCT will assist to gain a better understanding about the release kinetics of 3D printed dosage forms. However, it is evident that this formulation mitigates targeted release of SNEDDS containing API to the GI tract and that patient-centred medicines may be produced by this approach. A change of API and/or formulation composition can be done using this approach without affecting the printing process, which is not the case if the API is incorporated into the filament prior to printing. Furthermore, the fact that the API is spatially decoupled from the 3D printing of the filament limits the API exposure to elevated temperatures and is subsequently a desirable attribute of this approach. In conclusion this work shows that 3D printing can be used to produce patient-centred combinatorial drug products with different release and dosing properties depending on the patients’ need.

## Electronic supplementary material

Below is the link to the electronic supplementary material.

ESM 1 (PDF 3073 kb)

ESM 2 (MP4 47120 kb)

ESM 3 (MPG 33188 kb)

ESM 4 (MPG 41131 kb)

ESM 5 (MPG 56481 kb)

ESM 6 (MP4 9887 kb)

## ABBREVIATIONS

API: Active pharmaceutical ingredient
CAD: Computer-aided design
CBZ: Carbamazepine
FDM: Fused deposition modelling
GI: Gastrointestinal
HPLC: High-performance liquid chromatography
o.d.: Outer diameter
PCL: Poly-ε-caprolactone
PLA: Polylactic acid
PVA: Polyvinyl alcohol
SD: Standard deviation
SNEDD: Self-nanoemulsifying drug delivery
SNEDDS: Self-nanoemulsifying drug delivery system
STL: Stereolithography
SWLI: Scanning white light interferometry
TPI: Terahertz pulsed imaging
USP: United states pharmacopeia
XOR-CAD: Subvolume of the CAD data which is not shared with the XμCT volume
XOR-XμCT: Subvolume of the XμCT data which does not overlap with the CAD model
XμCT: X-ray computed microtomography
*Y*: Vertical position
Ψ: Azimuth angle
