# Supplementary material for: Analysis of 3D Prints by X-ray Computed Microtomography and Terahertz Pulsed Imaging
Source: Pharm Res. 2016 Dec 21;34(5):1037–52. doi: 10.1007/s11095-016-2083-1 (PMC5382186; doi:10.1007/s11095-016-2083-1)
Supplement: Supplementary file 1 — (PDF 3073 kb) [file 11095_2016_2083_MOESM1_ESM.pdf]

Supplementary Information to

Analysis of microstructure of 3D printed patient-centred dosage forms using X-ray micro-computed tomography (XμCT) and terahertz pulsed imaging (TPI)

Daniel Markl[1], J Axel Zeitler[1], Cecilie Rasch[2], Maria Høtoft Michaelsen[2], Anette Müllertz[2], Jukka Rantanen[2], Thomas Rades[2], and Johan Bøtker*[2]

[1]Department of Chemical Engineering and Biotechnology, University of Cambridge, Cavendish Laboratory, JJ Thomson Avenue, Cambridge, CB3 0HE, UK
[2]Department of Pharmacy, University of Copenhagen, Universitetsparken 2, 2100 Copenhagen, Denmark

July 13, 2016

Abstract

In this supplementary section we provide more results from the X-ray micro-computed tomography (XμCT) and terahertz pulsed imaging (TPI) measurements of the 3D printed dosage forms.

I. Terahertz Pulsed Imaging

Fig. 1 illustrates the peak intensity of the one-compartmental samples. The peak intensity is referred to as terahertz electric field peak strength (TEFPS), which is defined as the magnitude of the reflection coefficient from the surface relative to the reference reflection from a mirror. This parameter is derived from the time-domain terahertz-waveform by

$$TEFPS = \frac{r_{as}}{E_0} \tag{1}$$

with

$$r_{as} = \frac{n_s - n_a}{n_a + n_s}\,.$$

r_{as} is the reflection coefficient measured via the time-domain terahertz waveform. E_0 is the intensity of the reference incident terahertz pulse. The peak intensity is thus strongly affected by the refractive index of the surface and can be used to analyse relative density changes of the surface.

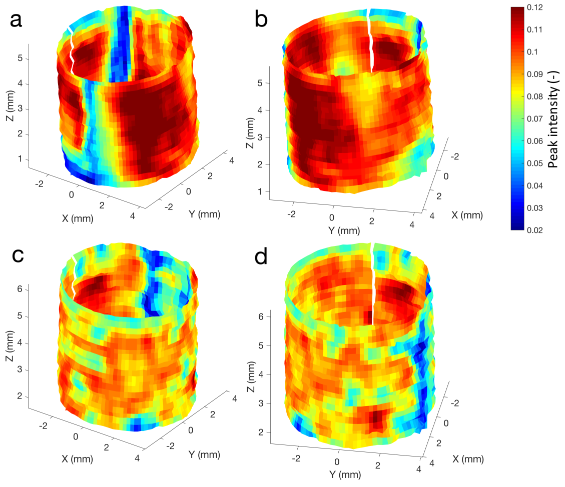

Figure 1: *Peak intensity from the time-domain terahertz waveforms of the 3D printed dosage forms. (a,b) and (c,d) are the renderings of two different orientations of the samples S01 and S02 (see Table I in manuscript for more information about these samples), respectively. The colourbar is valid for all subfigures.*

*johan.botker@sund.ku.dk

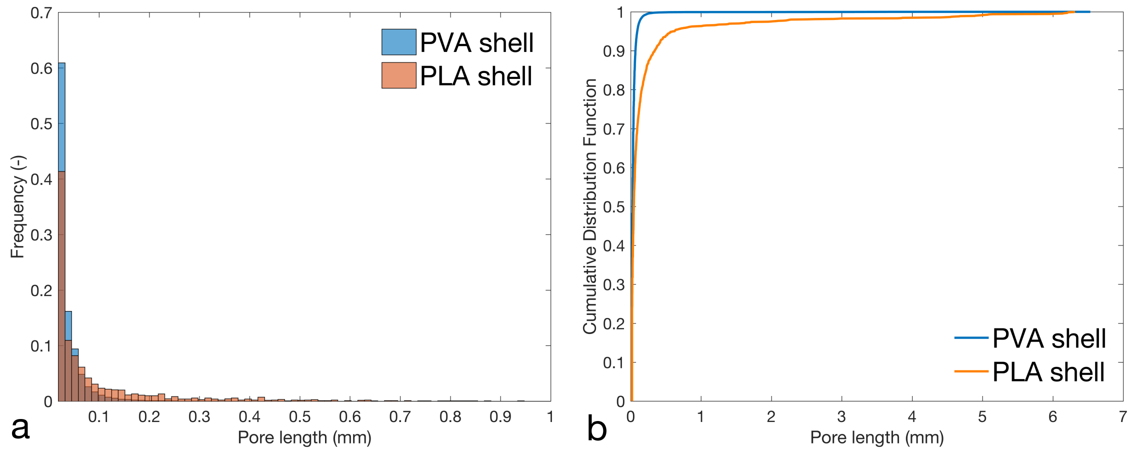

Figure 2: *Pore length distributions of the one-compartmental samples S01 (PVA shell) and S02 (PLA shell). The pore length is calculated of the pores extracted from the XµCT data of these samples (see Fig. 5 in manuscript). (a) Frequency distribution of the pore length. (b) Cumulative distribution of the pore length.*

Table 1: *Properties calculated from the XµCT and CAD data for sample S14 (compartmental sample filled with SNEDDS containing saquinavir in the outer compartment). The volume of the fill material is calculated by subtracting the average total XµCT volume of 1204.5 mm^3 of samples S04 – S06 from the total XµCT volume of the respective sample containing the liquid.*

		S14
XOR-CAD volume	mm^3	118.5
XOR-XµCT volume	mm^3	246.1
Total XµCT volume	mm^3	1244.8
XOR-XµCT volume / total CAD volume	%	22.0
XOR-CAD volume / total CAD volume	%	10.6
XµCT volume / CAD volume	%	111.4
Volume of fill material	mm^3	40.2

II. X-ray Micro-computed Tomography

i. Analysis of the Pore Structure

Fig 2 depicts the frequency distribution and cumulative distribution of the one-compartmental samples. These results indicate a clear difference between the PVA and PLA shells. The mean pore length is 0.04 ± 0.09 mm and 0.20 ± 0.72 mm for the samples S01 and S02, respectively. More properties of the pore structure can be found in Table II in the manuscript.

ii. Characterisation of Two-compartmental Samples Filled with SNEEDS

Fig. 3 visualises the XµCT rendering of two-compartmental samples filled with self-nanoemulsifying drug delivery system (SNEEDS) containing API. The SNEEDS formulation is the same is presented in section 2.1 in the manuscript except as Kolliphor® P 188 was replaced by Kolliphor RH 40. The filling material was thus a SNEDDS formulation consisting of soybean oil from Sigma-Aldrich (St. Louis, MO, US), Kolliphor® RH 40 from BASF (Ludwigshafen, Germany), Maisine 35-1

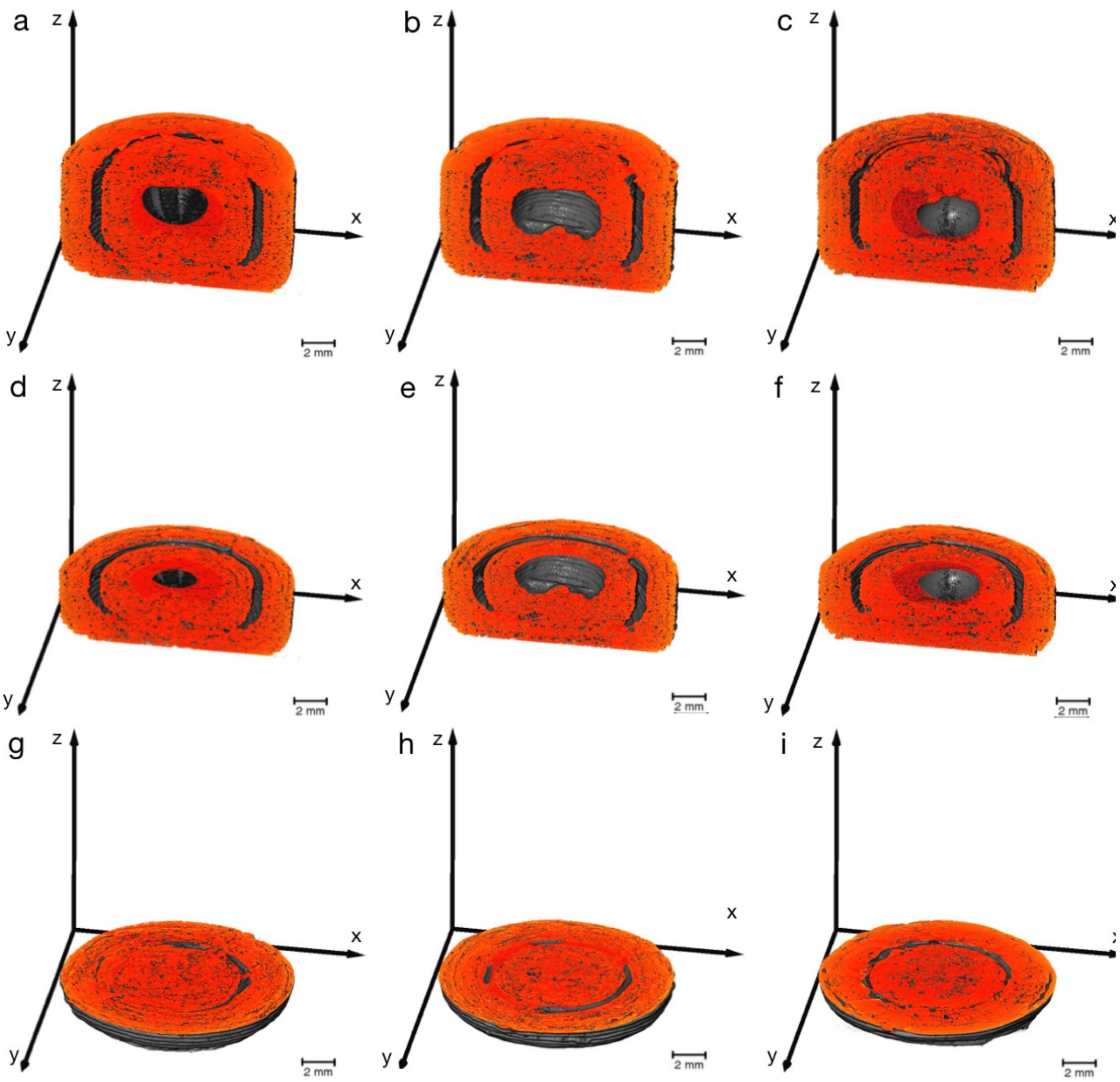

Figure 3: *Subvolumes of the two-compartmental geometries filled with a SNEDDS formulation generated from the XμCT data. The brighter regions correspond to the SNEDDS, which is noticeable in the outer and inner compartment (sample S13) in (a), (d) and (g), only in the outer compartment (sample S14) in (b), (e) and (h), and only in the inner compartment (sample S15) in (c), (f) and (i).*

from Gattefossé (Saint-Priest Cedex, France) and ethanol absolute from VWR international (Fontenay-Sous-Bois, France). The inner and outer compartment were filled with the SNEDDS formulation containing saquinavir or halofantrine, respectively. In the following the samples filled with SNEEDS in the inner compartment, outer compartment and both compartments are referred to as S13, S14 and S15, respectively. The volume of the SNEEDS formulation can be calculated by subtracting the average volume of the empty structures from the total XμCT volume of the filled samples (see 1). This was performed for the sample filled with SNEEDS in the outer compartment yielding a volume of 40 mm^3.

This is in good agreement with the results presented in the manuscript as well as with the actual loading amount of 50 µl of liquid.
